# Comparison of Characteristics of Suicide Attempts Registered in Urban and Rural Areas in China

**DOI:** 10.3389/fpsyt.2021.805324

**Published:** 2022-01-05

**Authors:** Lingling Li, Chunxu Liu, Yongsheng Tong, Jianlan Wu, Wei Zhou, Yi Yin, Mengjie Wu, Ruoyang Tong, Jing An

**Affiliations:** ^1^Beijing Suicide Research and Prevention Center, Beijing HuiLongGuan Hospital, Beijing, China; ^2^World Health Organization Collaborating Center for Research and Training in Suicide Prevention, Beijing, China; ^3^Peking University HuiLongGuan Clinical Medical School, Beijing, China; ^4^Beijing Tongzhou District Mental Health Hospital, Beijing, China; ^5^College of Foreign Languages, Capital Normal University, Beijing, China

**Keywords:** suicide attempt, characteristic, rural area, urban area, China

## Abstract

**Objective:** The objective of this study is to compare the characteristics of suicide attempts registered in general hospitals in urban and rural areas in China.

**Methods:** From January 2007 to December 2011, suicide attempts registered in hospitals in five rural counties and in the Beijing Municipality were included. Univariate and multivariate analysis were used to compare the characteristics of rural and urban suicide attempts in China.

**Results:** A total of 5,515 episodes of suicide attempts were included, 1,966 (35.6%) of them were from rural counties and 3,549 (64.4%) were from Beijing. Compared with urban counterparts, the rural suicide attempters had lower proportion of females (61.9% vs. 72.3%), more likely reporting previous suicide attempt history (56.9% vs. 16.4%), and staying in hospital for more than 1 day (81.5% vs. 44.6%). The most common methods of suicide attempts were pesticide ingestion in rural areas (52.1%) and taking medications in urban area (39.2%). Results of multivariate analysis indicated that suicide attempt registered in rural areas, pesticide ingestion, and previous suicide attempts history were associated with longer treatment in hospitals.

**Conclusions:** Suicide attempts registered in rural areas were different from those in urban areas in China. It is essential to improve the equipment and ability of medical resuscitation for pesticide ingestion in rural hospitals in China.

## Introduction

Suicide is a serious public health issue. Globally, more than 700,000 people died by suicide, and the age-standardized suicide rate in 2019 was 9.0 per 100,000 population (1). Furthermore, the number of attempted suicides is far more than that of suicide deaths. An estimate of more than 1,000,000 episodes of suicide attempts were medically treated in general hospitals in China (2).

History of suicide attempt is the one of the most powerful risk factors for subsequent suicide and/or suicide attempts (3, 4). Hence, understanding the characteristics of suicide attempts is helpful for making effective suicide prevention strategy (5). Most of the previous studies on suicide attempts in China occurred in the rural area (4, 6–9), yet little is known about the characteristics of suicide attempts in the urban area. Over the past 20 years, the overall suicide rates in China decreased dramatically, especially in rural China (10, 11). In the same periods, along with the rapid urbanization, the urban population drastically increased in China. In this scenario, suicide prevention in urban China would be our concern in the future (12). As yet, few literatures focused on the urban–rural differences of suicidal behaviors in China, although there were several studies that reported the characteristics of suicide attempts in urban China (13–16). It is reasonable to query whether suicide prevention strategies based on previous findings in rural China, e.g., limiting access to high lethal pesticides, could also be suitable and effective in urban China. To tailor more specific suicide prevention strategies for urban and rural areas, it is necessary to compare the characteristics of suicide attempts between the two different regions in China.

## Methods

### Settings and Participants

In the present study, we classified study sites into rural and urban areas, according to the dominant economic activities in each site. Due to several concerns (described below), we did not subdivide suicide attempters further according to their household registration (hukou system) information. First, making and implementation of suicide prevention strategies should consider socioeconomic and related characteristics of the involved regions (county, province, or country level), but it is unnecessarily altered for the hukou category of each individual. Although there were a few residents in urban areas engaged in farming, and a few residents in rural areas that did not engage in farming, it is impossible to change the overall characteristics and economic activities in corresponding regions. Second, the household registration does not accurately reflect the actual employment and living conditions of the suicide attempters. For example, the agricultural residents in Beijing are not necessarily engaged in agricultural production, and may also live in apartments in a high building, and the non-agricultural residents in rural counties may actually engage in agricultural activities. Finally, the collection of household registration information may be inaccurate. Due to various concerns (stigma, etc.), suicide attempters or their family members might provide inaccurate information.

For the rural areas, one agricultural county from each of the four provinces and one autonomous region in China were selected. The five sites in the present study were Yuncheng County in Shandong, Fengning County in Hebei, Chongzhou in Sichuan, Mei County in Shaanxi, and Kailu County in Inner Mongolia. The inclusion standard for these counties was “dominated by agricultural production,” which also referred to rural areas (17–19). In each of the aforementioned five counties, residents that engaged in farming accounts for about 80% of the total population. We have established the hospital registration system for suicides and attempted suicides in these five counties (20), by means of which we could obtain information about suicide attempts treated in the local general hospitals. All of the suicide attempts registered in general hospitals in these five counties were assigned to cases in rural areas.

Urban area refers to the densely populated area with a “non-agricultural economy, developed industry, and commerce as the mainstay” (17–19). According to the demographic report of Beijing in 2010, the whole Beijing Municipality is dominated by non-agricultural economic activity. The urban residents accounts for 86.0% of the overall permanent residents in the Beijing Municipality (21). Therefore, this study classified suicide attempts registered in general hospitals in Beijing as cases in urban area, regardless of the hukou category of the suicide attempters. In 2009, there were 272 general hospitals, including primary, secondary, and tertiary hospitals, in Beijing. Because of limited number of outpatient visits in the primary and secondary general hospitals, a systematic sampling method is used to sample one-third of the first- and second-level hospitals. However, all of the tertiary (the highest rank in China) general hospitals were included in the present study due to the large amount of outpatient visits. Finally, all attempted suicide cases treated in the emergency department of the included 110 general hospitals (56 primary, 19 secondary hospitals, and 35 tertiary hospitals) in the Beijing Municipality were recruited in this study (13).

Participants were suicide attempters medically treated in the hospitals due to their suicidal acts from January 1, 2007 to December 31, 2011. All the cases in this study were divided into two groups based on the location: rural group or urban group.

The inclusion criteria were ① patients who were medically treated due to their suicidal act and were registered in the hospital and ② age ≥10 years old. Exclusion criteria were as follows: ① Hospital health record clearly indicated that the case was accidental poisoning or injury. ② The suicide attempt was repeatedly registered by different hospitals within 72 h (it was regarded as one episode of suicide attempt, and only one of the records was kept), ③ The hospital health record showed that the patient died by the index suicidal act. The study was approved by the IRB of Beijing Huilongguan Hospital. A written consent form was waived because the data analysis was based on registered data only.

### Measurement

Self-complied suicide attempt registration form was used to record relevant information about patients and their suicidal behaviors. The information includes hospital name, code of the name of the patient, gender, age, home address, self-harm category (suicide attempt or accidental injury/poisoning), whether the patient died by the suicidal behavior, specific method of suicide attempt, history of previous suicide attempts, the time (month) the suicide attempt occurred, duration of the treatment in hospital, and specific treatment methods.

Whether the case was suicidal behavior or accidental poisoning or injury was determined briefly by a trained doctor. The judgment was based on comprehensive information, including the process of self-poisoning or injury, around psychosocial environment of the self-harm, etc. (20). Attempted suicide was defined as self-harm with a non-fatal outcome and an intentional killing of oneself (22).

### Statistical Analysis

For those patients with the same name, age, sex, and address, we considered them as a duplicated case of the same patient. If the time interval between two or more duplicated records of same case is shorter than 72 h, it would be recorded as one episode of suicide attempt. If the time interval is 72 h or longer, it would be regarded as repeated suicide attempts of the same patient.

The statistical analyses were run by SPSS 25.0. Descriptive analysis, independent *t*-test, chi-square test, and logistic regression analysis were conducted. Demographic characteristics (such as gender and age) and specific characteristics of attempted suicide were compared between rural and urban groups. Multivariate logistic regression was used to analyze the correlates of duration of staying in hospitals for treatment. Significant level of α was set as <0.05, and a two-sided test was used.

## Results

### Characteristics of the Two Groups of Suicide Attempts

In total, 7,091 cases of self-harm were registered in this study of which 1,353 cases were identified as accidental self-poisoning or injury, 73 cases were duplicated registrations of the same self-harm, and 150 cases died of the self-harm. Among the 150 deaths, 41 cases were located in Beijing and the other 109 cases in rural areas. The case fatality of the registered self-harm was statistically significantly higher in rural areas than in urban areas (Beijing) (χ^2^ = 86.21, *p* < 0.01). After these cases were excluded, a total of 5,515 episodes of suicide attempts were included in the final data analysis of the present study. Among them, 1,966 episodes of suicide attempts (35.6%) occurred in the rural areas. Of them, 254 (4.6%) occurred in Yuncheng County, Shandong, 103 (1.9%) in Fengning County, Hebei, 945 (17.1%) in Chongzhou, Sichuan, 572 cases (10.4%) in Mei County, Shaanxi, and 92 cases (1.7%) in Kailu County, Inner Mongolia. The other 3,549 episodes of suicide attempts (64.4%) occurred in the urban area (Beijing).

Suicide attempters in rural areas were older [(35.88 ± 15.67) vs. (34.42 ± 15.07), t = 3.39, *p* < 0.01] and less likely being female (61.9% vs. 72.3%, χ^2^ = 62.32, *p* < 0.01) than those in the urban area. Nearly half of the suicide attempters in the rural areas (48.1%) and in the urban areas (59.1%) were aged 15–34 years old (see Table 1 for details).

**Table 1:** Comparison of demographic variables and characteristics of suicide attempts registered in rural and urban hospitals.

**Variables**	**Rural areas *n* (%)**	**Urban area *n* (%)**	**χ^2^/*t***	***p-*Value**
Gender			62.32	<0.01
Female	1,209 (61.9)	2,554 (72.3)		
Male	743 (38.1)	980 (27.7)		
Age (years old)			72.41	<0.01
10–14	50 (2.6)	37 (1.1)		
15–34	939 (48.1)	2,062 (59.1)		
35–54	712 (36.4)	1,006 (28.8)		
55 and above	253 (12.9)	383 (11.0)		
Suicide method			972.90	<0.01
Pesticide	1,024 (52.1)	534 (15.0)	856.29	<0.01
Overdosing medication	374 (19.0)	1,391 (39.2)	236.54	<0.01
Other poisonings	391 (19.9)	1,125 (31.7)	88.54	<0.01
Knife/rope/drowning	84 (4.3)	44 (1.2)	51.33	<0.01
Other methods (CO, excessive drinking, etc.)	93 (4.7)	455 (12.8)	92.53	<0.01
Duration of treatment in hospital			658.69	<0.01
Leave the hospital on the same day	330 (18.5)	1,938 (55.4)		
Staying in hospital >1 day	1,457 (81.5)	1,560 (44.6)		
Previous suicide attempt^*^			337.98	<0.01
0	846 (43.1)	572 (83.6)		
1	719 (36.6)	86 (12.6)		
2 or more	398 (20.3)	26 (3.8)		
In-hospital treatment^*^				
Gastric lavage	1,228 (62.5)	573 (83.9)	106.99	<0.01
Intramuscular injection	1,496 (76.1)	134 (19.6)	683.01	<0.01
Intravenous infusion	1,745 (88.8)	629 (92.1)	6.06	0.01
Hemostasis	67 (3.4)	46 (6.7)	13.74	<0.01
Artificial breathing	11 (0.6)	6 (0.9)	0.81	0.37
Other	95 (4.8)	52 (7.6)	7.48	0.01

* *Only 684 of the suicide attempts registered in urban hospitals have recorded information of the two variables*.

The most common method of suicide attempts in rural areas was pesticide ingestion (52.1%), and such proportion was higher in the rural areas than that in the urban area (15.0%). The main methods of suicide attempts in the urban area were medication overdose (39.2%) and other poisonings (31.7%), and the proportions of both of the two methods were higher than those in the rural areas (see Table 1 for details).

Only 684 episodes of suicide attempts in the urban area recorded whether there was a previous suicide attempt. The proportion of reporting a history of suicide attempt was substantially lower in the urban area (16.4%) than that in the rural areas (56.9%), and it reached statistical significance (χ^2^ = 334.98, *p* < 0.01). Suicide attempters in the rural areas were more likely reporting a history of two or more episodes of previous suicide attempts than those in the urban area (see Table 1 for details).

**Figure 1:**
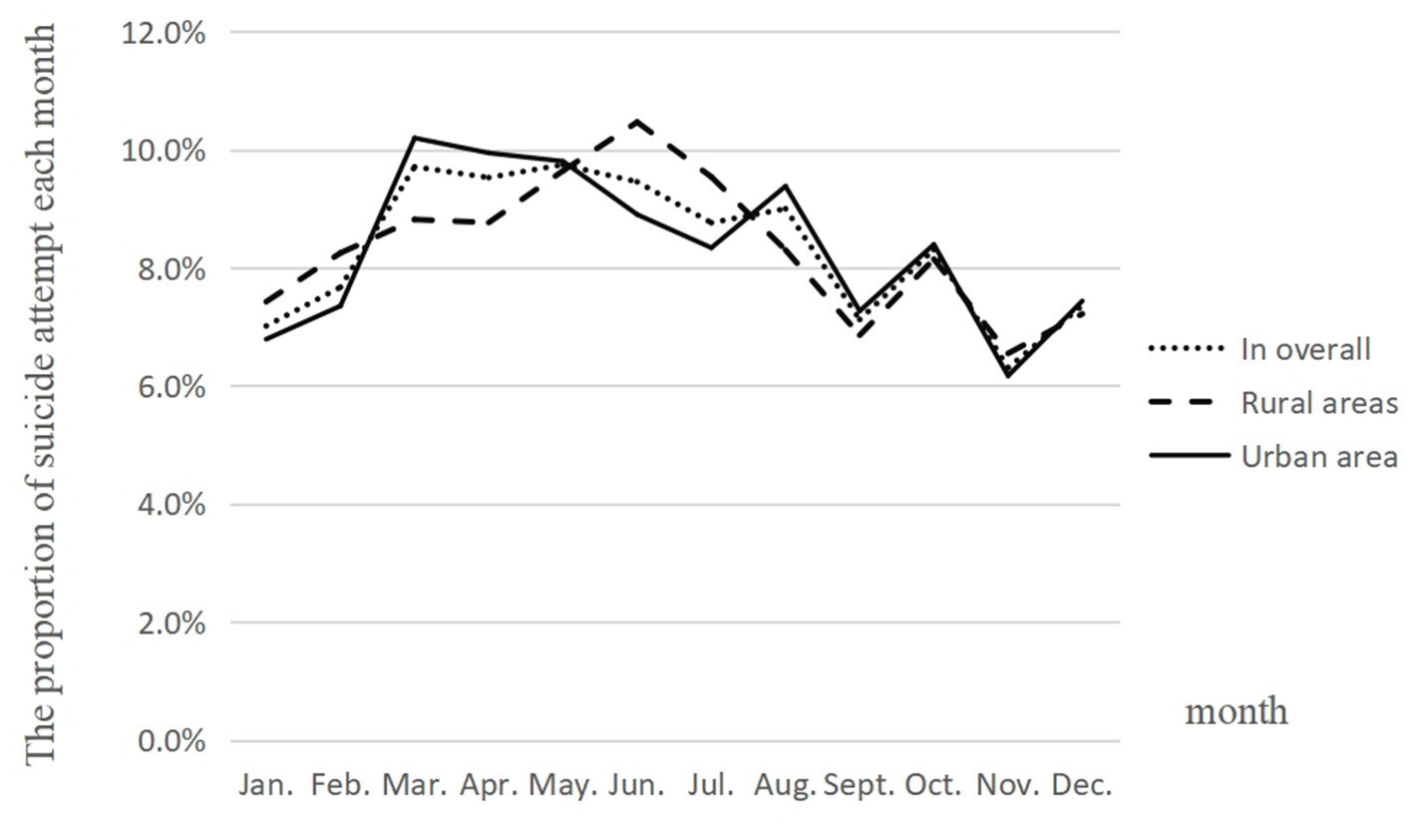
Monthly distribution of suicide attempts registered in the rural and urban areas.

The monthly distribution of attempted suicides in both of the urban and rural areas ranged from 6.2 to 10.5% (Figure 1). The occurrence of suicide attempts varied across 12 months and reached statistical significance (χ^2^ = 104.89, *p* < 0.01). In the rural areas, suicide attempts more likely occurred in May to July than in other months, while in the urban area, suicide attempts more likely occurred in March to May than in other months. There was no statistically significant difference in the monthly distribution of attempted suicides between the rural and urban areas (χ^2^ = 14.02, *p* = 0.230).

### Treatments in Hospital Due to Suicide Attempts

Gastric lavage, intramuscular injection, and intravenous infusions were the most common treatment methods for suicide attempters in hospitals in both the rural and urban areas. However, the proportion of gastric lavage and intravenous infusion in urban hospitals were statistically significantly higher than that in rural hospitals, while the proportion of intramuscular injection in rural hospitals was substantially higher than that in urban hospitals (see Table 1 for details).

More than half of the suicide attempts were hospitalized for more than 1 day. The suicide attempts treated in the rural hospitals were more likely staying for more than 1 day in the hospital than those treated in urban hospitals (81.5% vs. 44.6%), and the difference was statistically significant (χ^2^ = 658.69, *p* < 0.01). The duration of staying in the hospital for treatment was associated with the methods of suicide attempts (χ^2^ = 334.07, *p* < 0.01). Three-quarters of the suicide attempts ingesting pesticides, using a knife/rope, or drowning were treated in the hospital for more than 1 day. Slightly more than half of the suicide attempts with medication overdose spent more than 1 day in the hospital for treatment, while a little bit lower than half of the suicide attempts using other poisons or others or unknown methods were staying in the hospital for more than 1 day (see Table 2 for details).

**Table 2:** Duration of being treated in the hospital for various suicide methods.

	**Leave the hospital on the same day *n* (%)**	**Staying in hospital >1 day *n* (%)**	**χ^2^**	***P***
Pesticides (1,418)	335 (23.6)	1,083 (76.4)	294.33	<0.01
Overdosing medication (1,731)	827 (47.8)	904 (52.2)	28.84	<0.01
Other poisonings (1,502)	781 (52.0)	721 (48.0)	70.67	<0.01
Knife/rope/ drowning (95)	23 (24.2)	72 (75.8)	13.81	<0.01
Other methods (CO, excessive drinking, etc., 539)	302 (56.0)	237 (44.0)	42.15	<0.01

### Multivariate Logistic Regression Analysis of Treatment Duration

Results of multivariate logistic regression analysis indicated that age (*p* < 0.05), selected methods for suicide attempts (*p* < 0.01), rural/urban areas (*p* < 0.01), history of previous suicide attempts (*p* < 0.01), and treatment methods (*p* < 0.01) were associated with treatment duration (see Table 3). Suicide attempts that occurred in the urban area (OR = 0.20) and treated with gastric lavage (OR = 0.37) were less likely to stay in the hospital for more than 1 day; however, suicide attempt with pesticide ingestion (OR = 2.76), with a history of previous suicide attempts (OR = 2.42), treated using intramuscular injection (OR = 2.52) and intravenous infusions (OR = 3.03) were more likely to stay in hospitals for more than 1 day than their reference groups.

**Table 3:** Correlates of duration of being treated in hospitals due to suicide attempts, using multivariate logistic regression analysis.

**Variables**	**β**	***Wals***	***OR***	***95% CI***	***p-Value***
**Gender**
Male	0.10	0.64	1.10	0.87–1.40	0.42
**Age (years old)**
55 and above			1.00	–	–
10–14	−0.48	1.07	0.62	0.25–1.53	0.30
15–34	−0.44	5.88	0.65	0.45–0.92	0.02
35–54	−0.38	4.13	0.68	0.47–0.99	0.04
**Suicide method**
Other methods (CO, excessive drinking, etc.)			1.00	–	
Pesticide	1.01	15.31	2.76	1.66–4.58	<0.01
Overdosing medication	−0.31	1.37	0.74	0.44–1.23	0.24
Other poisonings	−0.48	3.12	0.62	0.37–1.05	0.08
Knife/rope/drowning	0.17	0.19	1.18	0.55–2.53	0.67
**Area**
Urban	−1.63	145.15	0.20	0.15–0.26	<0.01
**History of previous suicide attempts**
Yes	0.89	47.70	2.42	1.89–3.11	<0.01
**In-hospital treatment**
Gastric lavage	−1.00	41.05	0.37	0.27–0.50	<0.01
Intramuscular injection	0.92	49.90	2.52	1.95–3.25	<0.01
Intravenous infusion	1.11	30.11	3.03	2.04–4.50	<0.01

## Discussion

Although there were several studies focused on suicide attempts that occurred in rural or urban areas in China, a few of the studies compared the characteristics of suicide attempts from the two areas with different socioeconomic environments. In the present study, the characteristics of suicide attempts between the rural and urban areas were substantially different in several aspects. The results showed that patients who received medical treatment in hospitals due to non-fatal suicidal behavior were mainly aged 15–34 in rural and urban areas. The suicide attempters in the rural areas were less likely as female and more likely reporting a history of previous suicide attempts than their counterparts in the urban area. Pesticide ingestion was the most common method of suicide attempts in the rural areas, while medication overdose and using other poisons were frequent methods of suicide attempts in the urban area. In terms of treatments in hospitals due to suicide attempts, gastric lavage and intravenous infusion were more frequently used in the urban area, and intramuscular injection was more frequently used in the rural areas. Rural suicide attempts were more likely treated for more than 1 day than urban suicide attempts, and such duration of treatment was associated with whether the suicide attempt occurred in the rural areas, pesticide ingestion, treatment methods, and history of previous suicide attempts.

Our findings indicated that the the 15- to 34-year-old age group accounts for most of the attempted suicide cases treated in general hospitals in both the urban and rural areas. Many previous studies have also reported that the majority of suicide attempters were teenagers (14, 23). The results of a WHO multicenter collaborative study indicated that more than half of the centers found that the highest incidence of suicide attempts was among people aged 25–34; meanwhile, the lowest incidence of suicide attempts was among those of 55 years and older (23, 24). Therefore, reducing suicide attempts among adolescents and young adults should be an important target of suicide prevention.

Consistent with previous studies (14, 25–28), results in our study also identified that females account for two-thirds of hospitalized suicide attempters. However, our findings indicated that the proportion of female suicide attempters in the rural areas (61.1%) was lower than that in the urban area (75.7%). The possible explanation is the different lethality of suicide methods used by the rural and urban women. Previous studies reported that the case fatality of suicidal acts using pesticide ingestion was 13%, while that of other methods such as overdosing medication and cutting was 4–7% (2). In urban China, since pesticides are not easily available, suicidal acts using pesticide ingestion are rare (2). It suggested that there are a considerable number of rural young females who died by suicide due to using highly lethal pesticides and the failure of treatment. That is, the relatively lower proportion of female suicide attempters in rural areas would be partly attributed to more “failed suicide attempts” in rural females.

Although China suicide rates decreased dramatically in the last 2 decades (11), the rural–urban differences in suicide rates are still outstanding (6). Suicide method selection might contribute to the differences. In the present study, pesticide ingestion was the most common method of suicide attempt in the rural areas; however, in the urban area, overdosing medication and using other poisons were frequently used methods of suicide attempt. Selection of suicide methods are related to the acceptability of suicidal behavior and the availability of suicidal methods (29). Due to the high availability, pesticide ingestion is the most common method of suicide in rural China (30), meanwhile, the proportion of suicides by taking medications has greatly increased in urban China (31). The other reason of frequent overdosing medication or using other poisons as suicide method is the belief that using such method may not only induce less pain to their bodies but also keep their bodies intact after death (15).

Given the rural–urban differences of suicide methods identified in our study and previous studies (14), along with the rapid progress of urbanization in China, we should make urban-specific suicide prevention strategy of limiting access to suicide methods. For instance, the permitted amounts of medications should be strictly limited in the prescription or selling in pharmacies (for over-the-counter medications), and family members of patients with suicidal ideation should strengthen the safe storage and management of prescribed medications. Certainly, reducing toxicity of commonly used pesticides to humans, limiting access to highly lethal pesticides, and improving resuscitation ability for poisoning by pesticides in rural hospitals should also be the main elements of suicide prevention strategies in rural China (2, 32).

More than four in five rural suicide attempts and nearly half of urban suicide attempts stayed in the hospital for treatment for more than 1 day; however, gastric lavage was less used, and intramuscular injection was more frequently used in rural hospitals than in urban hospitals. Several factors might associate with the duration of treatment in hospitals. After demographic variables were adjusted, suicide attempts by pesticide were more likely to stay in hospitals for more than 1 day than that by other methods (OR = 2.76). Pesticide poisoning may lead to more serious physical injuries, especially for highly toxic pesticides, such as paraquat. Treatment method is another predictor of the duration of staying in the hospital. Gastric lavage was associated with less treatment duration (OR = 0.37); however, intramuscular injection (OR = 2.52) and intravenous infusion (OR = 3.03) were associated with longer treatment duration. The treatment method, to some extent, reflects severities of body injuries due to the suicide attempts. Gastric lavage might be sufficient enough to rescue medication overdose; however, for resuscitation of organophosphorus pesticide poisoning, intramuscular injection of atropine and/or muscarinic antagonist drugs are necessary (33), in addition to the gastric lavage. Last and importantly, after adjusting for factors mentioned above, whether suicide attempts occurred in the rural areas was also associated with treatment duration. Suicide attempts registered in the urban area were less likely treated in hospitals for more than 1 day (OR = 0.20). Considering limited equipment and drugs in rural hospitals, especially in those low- and middle-income countries, improvement of resuscitation ability for pesticide poisoning in rural hospitals is a key component of the suicide prevention strategies (33).

Additionally, our findings also found that suicide attempters in the rural areas were more likely reporting a history of previous suicide attempts than that in the urban area. Previous studies (7) have shown that the history of attempted suicide is an independent risk factor for subsequent suicidal behavior. Those with a previous history of attempted suicide have long-term negative life events and chronic psychological stress. Most of them suffer from mental illness (especially depression and schizophrenia). Therefore, it is essential to strengthen post-intervention for suicide attempters in the rural areas (8).

In the present study, we did not find a rural–urban difference on the monthly distribution of suicide attempts, although occurrence of suicide attempts varied across 12 months. However, a previous study in Hebei Province (9) found that 51.0% of suicidal behavior using pesticide occurred in the summer (June to August), and especially in March and May. Another study on prehospital rescued patients due to suicidal acts also reported that suicide attempts occurred less in February and December (16). Thus, further studies are needed to explore the time distribution of suicide attempts.

There were several limitations of the present study. First, most of the research sites were located in the north of China. Thus, the findings could not be generalized to the other regions of China. Second, although most of the hospitals in Beijing and the five rural counties were selected in the present study, some hospitals in these areas had not been included in the registration system. In addition, a few suicide attempt cases might not be registered. The slight sampling bias would also limit the representativeness of the sample in our study. Third, the present study only recruited attempted suicides, our findings cannot be generalized to suicide deaths, considering the characteristics of suicide attempts were different from suicide death. Fourth, there were also differences in the gender and age distributions of population that lived in urban and rural China, the differences on demographic variables between suicide attempters in the rural and urban areas needs further exploration. Fifth, the suicide attempt registration form used in this study is self-complied. Although it contains most information related to suicidal behavior, it is not yet standardized. Finally, not all suicide attempters would go to the hospital seeking treatment, thus, untreated suicide attempts were not included in this study.

In conclusion, the findings of the present study indicate that the characteristics of suicide attempts between the rural and urban areas were substantially different. For example, pesticide ingestion was the most common method of suicide attempt in the rural areas, while medication overdose and using other poisons were the frequent methods of suicide attempt in the urban area. Therefore, suicide prevention strategies should be tailored according to specific conditions in the urban and rural areas in China.

## Data Availability Statement

The raw data supporting the conclusions of this article will be made available by the authors, without undue reservation.

## Ethics Statement

The studies involving human participants were reviewed and approved by the IRB of Beijing HuiLongGuan Hospital. Written informed consent to participate in this study was provided by the participants' legal guardian/next of kin.

## Author Contributions

LL, CL, and YT designed the study. LL wrote the initial draft of the article. JW, WZ, and RT assisted in the preparation of the article. YT and JA contributed to the data collection. YT, YY, and MW contributed to the interpretation of the data and reviewed the article. All authors approved the article and agreed to be accountable for all aspects of the work in ensuring that questions related to the accuracy or integrity of any part of the work are appropriately investigated and resolved.

## Funding

This research was partly supported by the Beijing Hospitals Authority Clinical Medicine Development of Special Funding Support (ZYLX202130) and the National Natural Science Foundation of China (82071546).

## Conflict of Interest

The authors declare that the research was conducted in the absence of any commercial or financial relationships that could be construed as a potential conflict of interest.

## Publisher's Note

All claims expressed in this article are solely those of the authors and do not necessarily represent those of their affiliated organizations, or those of the publisher, the editors and the reviewers. Any product that may be evaluated in this article, or claim that may be made by its manufacturer, is not guaranteed or endorsed by the publisher.
